# Growth Mechanisms of ZnO Micro-Nanomorphologies and Their Role in Enhancing Gas Sensing Properties

**DOI:** 10.3390/s21041331

**Published:** 2021-02-13

**Authors:** Ambra Fioravanti, Pietro Marani, Sara Morandi, Stefano Lettieri, Mauro Mazzocchi, Michele Sacerdoti, Maria Cristina Carotta

**Affiliations:** 1 Istituto di Scienze e Tecnologie per l’Energia e la Mobilità Sostenibili (CNR–STEMS), Via Canal Bianco 28, 44124 Ferrara, Italy; pietro.marani@stems.cnr.it; 2 Dipartimento di Chimica, Università di Torino, Via P. Giuria 7, 10125 Torino, Italy; sara.morandi@unito.it; 3 Istituto di Scienze Applicate e Sistemi Intelligenti “E. Caianiello” (CNR-ISASI), Complesso Universitario di Monte S. Angelo, Via Cupa Cintia 21, 80126 Napoli, Italy; stefano.lettieri@isasi.cnr.it; 4 Istituto di Geoscienze e Georisorse (CNR-IGG), Via G. La Pira 4, 50121 Firenze, Italy; mauro.mazzocchi@igg.cnr.it; 5 Dipartimento di Fisica e Scienze della Terra, Università di Ferrara, Via Saragat 1, 44122 Ferrara, Italy; michele.sacerdoti@unife.it

**Keywords:** ZnO nanomorphologies, ε-Zn(OH)_2_, growth mechanisms, pseudomorphism, chemoresistive gas sensors, thick films, VOCs detection

## Abstract

Zinc oxide (ZnO) is one of the main functional materials used to realize chemiresistive gas sensors. In addition, ZnO can be grown through many different methods obtaining the widest family of unique morphologies. However, the relationship between the ZnO morphologies and their gas sensing properties needs more detailed investigations, also with the aim to improve the sensor performances. In this work, seven nanoforms (such as leaves, bisphenoids, flowers, needles, etc.) were prepared through simple wet chemical synthesis. Morphological and structural characterizations were performed to figure out their growth mechanisms. Then, the obtained powders were deposited through screen-printing technique to realize thick film gas sensors. The gas sensing behavior was tested toward some traditional target gases and some volatile organic compounds (acetone, acetaldehyde, etc.) and compared with ZnO morphologies. Results showed a direct correlation between the sensors responses and the powders features (morphology and size), which depend on the specific synthesis process. The sensors can be divided in two behavioral classes, following the two main morphology kinds: aggregates of nanocrystals (leaves and bisphenoids), exhibiting best performances versus all tested gases and monocrystal based (stars, needle, long needles, flowers, and prisms).

## 1. Introduction

Sensors have become of crucial importance in several aspects of our daily life, allowing us to monitor physical, chemical, biological, and environmental parameters [1,2,3]. Small dimensions, low cost and consumption, easy use, wearability, and light weight are required to use sensors in a great number of new applications [4].

Metal oxides (MOX) materials are major players in the field of chemical sensing. Well-established methods, including wet chemistry [5,6] and different physical depositions techniques [7,8,9,10,11] are available for preparing MOX nanoparticles and nanostructured films, allowing to tailor their morphology for targeted applications in chemical sensing [12,13,14,15].

Several MOX semiconductors are known to exhibit gas-sensitive fluorescence and optical responses, permitting their use as optochemical sensors as fluorescent films [16,17] or by integrating them in photonic devices such as optical fibers [18] or photonic crystals [19,20]. However, the most relevant application of MOXs in chemical sensing is in realization of chemiresistors. Air quality monitoring in indoor/outdoor environment [21,22,23,24,25], clinical disease diagnosis [26], food quality monitoring [27], diagnostics for industrial systems [28], and safety control devices, [29] are important examples of such applications.

MOX properties depend significantly on their defect composition and nanocrystals morphology, as discussed in this work. To date, a lot of efforts are addressing to improve sensing, selectivity, and reliability properties of MOX gas sensors [30,31]. In this respect, the functional material preparation, with particular emphasis to nanoscale processes, still remains the key issue to obtain oxides with tunable physical–chemical properties that lead to the fabrication of sensor devices with specific functionality [32,33].

In this context, zinc oxide (ZnO) has a special role, as it is both a chemoresistive material and a photonic material, exhibiting a strong excitonic photoluminescence that is not thermally quenched due to its large exciton binding energy. Similarly to other widely MOXs used for chemical sensing (e.g., TiO_2_, SnO_2_, and WO_3_), ZnO is a n-type semiconductor and certainly a well-performing material for sensor devices the most promising oxide primarily due to its chemical and morphological stability and its high gas sensitivity. In fact, ZnO has been successfully employed to detect various gases, such as H_2_, NO_2_, O_2_, H_2_S, C_2_H_6_O, and NH_3_ [34,35,36,37,38,39,40,41,42]. Furthermore, ZnO exhibits a strong aptitude to be grown in a wide diversity of 0-, 1-, 2-, and 3-dimensional nanomorphologies with different functional properties. ZnO can be prepared through a great number of methods (such as chemical or physical vapor deposition [43], wet chemical methods [44], etc.), obtaining a wide family of nanostructures. This aptitude is of central importance in gas sensors because the morphology directly influences the sensors’ performances.

Although literature reported several previous studies [34,45,46,47,48] and reviews of ZnO nanostructures for gas sensing [49,50,51], there are few systematic investigations on the dependence of ZnO detection performances on their morphology. However, this is a topic that deserves dedicated attention, as the morphology of the samples is no less than other important parameter (along, for instance, the defectivity, the eventual extrinsic doping and the specific surface area) that define their gas-sensing ability.

The aim of the present work is to focus on the correlation between morphology and sensing properties of ZnO structures, investigating how the former influences the response to the target analytes. In particular, we carried out an analysis of conductance response on seven types of ZnO with different morphologies towards several different analytes (acetone, acetaldehyde, formaldehyde, isoprene, toluene, ethanol, and ammonia). The samples were obtained through wet chemical synthesis methods, which we choose because they represent a versatile and powerful technique for growing ZnO nanostructures [52]. Particular attention was paid to analyze the growth mechanisms from which the final morphology depends. Finally, thick film gas sensors were realized starting from the ZnO synthesized nanopowders by means of the screen printing technique, such method permitting to separately study and optimize both the functional material synthesis and the film deposition [53]. Electrical and gas sensing properties were examined and compared with the powder morphology. The results confirm a direct correlation between the sensors responses and the powders’ features.

## 2. Materials and Methods

### 2.1. Synthesis of ZnO Morphologies

**Figure 1:**
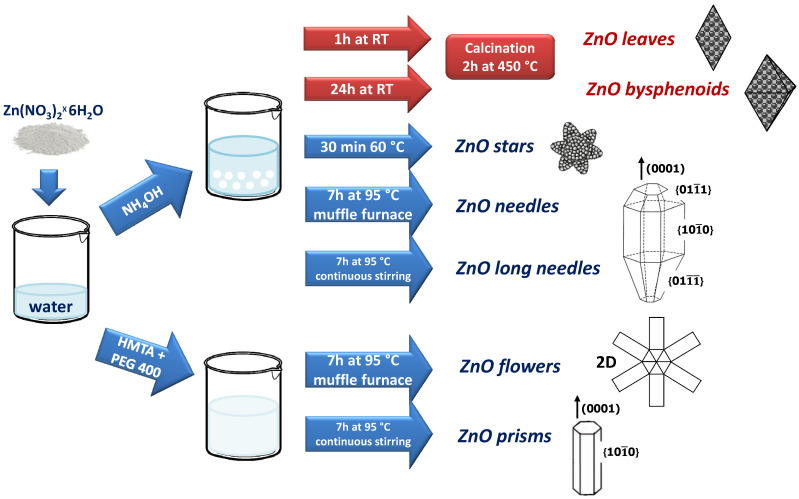
Schematic representation of the synthesis procedures.

All ZnO powders were obtained by dissolving zinc nitrate hexahydrate (Zn(NO_3_)_2_ × 6H_2_O, >99.0%, Sigma-Aldrich, used as received by the Merck Group supplier, Milan, Italy) in water solution and using a weak base (ammonium hydroxide, 28% Carlo Erba Reagents or hexametilenetetramine–HMTA, Sigma-Aldrich) to catalyze the hydrolysis. The different morphologies were achieved modifying the synthesis conditions, as schematically represented in the Figure 1.

*Leaves and Bisphenoids* were synthesized starting from a 0.05 M water solution of Zn(NO_3_)_2_ × 6H_2_O. Water diluted ammonium hydroxide (28%, Carlo Erba Reagents) was added to reach a pH of 10. The solution was aged for 1 or 24 h at room temperature to obtain leaves and bisphenoids, respectively. In both cases, the white precipitate was filtered by gravity, washed with water and diethyl ether, afterwards it was dried in air for 12 h at 100 °C in oven, and finally calcined for 2 h at 450 °C.

*Stars* were achieved following the same route of leaves and bisphenoids heating the solution at 60 °C and maintaining it at that temperature for 30 min. The white ZnO precipitate was filtered by gravity, washed with water and diethyl ether, and afterwards it was dried in air for 12 h at 100 °C in oven.

*Needles and long needles* were obtained following the same route of leaves and bisphenoids, thereafter submitting the mixture to a thermal treatment at 95 °C for 7 h in a muffle furnace for needles, or using a hotplate under continuous stirring for long needles. In both cases, the white ZnO precipitate was filtered by gravity, washed with water and diethyl ether, and after it was dried in air for 12 h at 100 °C in oven.

*Flowers and prisms* were prepared from a water solution of Zn(NO_3_)_2_ × 6H_2_O 0.1 M. A second solution of HMTA 0.05 M was prepared and added under continuous stirring to the first one. Then, a small quantity of polyethylene glycol (PEG, MW = 400), 0.1 mol% with respect to Zn(NO_3_)_2_ × 6H_2_O, was introduced. The final solution subjected to a thermal treatment at 95 °C for 7 h in a muffle furnace in the case of flowers or using a hotplate under continuous stirring for prisms. In both cases, the white ZnO precipitate was filtered by gravity, washed with water and diethyl ether, and afterwards it was dried in air 12 h at 100 °C in oven.

The whole system of the described synthesis procedure has been optimized than that reported in a previous work [54] and, to obtain a repeatable process, the preparations were repeated more than 5 times. A yield of about 90% was reached for all procedures except for leaves and stars that resulted as 46% and 73%, respectively.

### 2.2. Morphological, Textural and Structural Characterizations

The morphology of the precursor of ZnO, of ZnO powders, and of the respective sensing films was studied using a Carl Zeiss Sigma Field Emission Scanning Electron Microscope (FE-SEM).

The Brunauer–Emmett–Teller (BET) method was used to estimate the specific surface area of the samples. It was applied to the adsorption/desorption isotherms of N_2_ at 77 K obtained with a Micromeritics ASAP 2010 physisorption analyzer.

X-ray diffraction (XRD) analysis was performed by means of a vertical Philips PW 1830 diffractometer (PANalytical, formerly Philips Analytical, Almelo, the Netherland) that works with Bragg-Brentano geometry. It was used Cu Kα radiation (40 kV, 30 mA) and the diffraction patterns were collected from 15° to 90° (2θ) with steps of 0.02° and 10 s of dwell time. The Rietveld refinement was adopted to estimate the unit cell parameters and carried out using FullProf program (release 2011) [55]. The Scherrer’s formula was applied onto the three main diffraction peaks of each diffractogram and the mean values were calculated obtaining the average crystallite size [56].

### 2.3. Thick Film Gas Sensors Realization

The thick films were deposited through screen printing technique, which offers large production capability, low cost, moreover allowing to work on the different manufacturing steps of the sensors, separately. ZnO powders were mixed with a mixture of organic vehicles and a minimum quantity of a glass frit to obtain viscous pastes suitable to be printed. Alumina square plates were used as substrates, with sides of 2.54 mm and thickness of 0.25 mm. On the front side, interdigitated gold electrical contacts serve to record the electrical responses of the sensing layers, while, on the opposite side, a platinum heater element has a function to heat the sensor at the proper working temperature.

The sensing layers are deposited with a thickness usually ranging from 15 to 30 μm, afterwards they underwent to a double thermal process: drying (at about 150 °C) and firing (at temperatures ranging between 650 and 850 °C). During the thermal process, the organic vehicle is totally removed, the glass frit melts and promotes a controlled powder sintering and the adhesion of the films to the substrate; at the same time the films reach the desired microstructure and electrical stability.

### 2.4. Electrical Characterizations

To test the electrical properties of the ZnO thick film sensors, they were lodged in a sealed test chamber kept at a constant temperature of 23 °C to exclude the temperature influence on sensors behavior during the measurements [24]. All tests were carried out using synthetic air as carrier gas in dry conditions keeping a constant flow rate of 0.5 L/min. To test the sensors, a typical DC (direct current) conductance measurement through an electronic circuitry with constant voltage over the sensor was adopted, ensuring a known and stable measuring potential.

Conductance measurements toward temperature (Arrhenius plots) were carried out in different atmospheres between about 300 and 900 K at the heating rate of about 3 K/min. To determine the most suitable morphology to detect a certain gas, dynamic measurements of conductance in presence of a large series of tested gases (acetaldehyde, acetone, ethanol, formaldehyde, toluene, isoprene, and ammonia), at operating temperatures varying from 300 to 550 °C were performed. Moreover, to better recognize the sensor responses, the conductance was normalized to 1. In such a way, the response was defined as the ratio between the conductance in gas and the conductance in air.

## 3. Results and Discussion

### 3.1. Morphological, Textural, and Structural Characterizations

The common and stable zinc oxide phase at ambient conditions is the hexagonal wurtzite structure, which consists of alternating planes composed of tetrahedrally coordinated O^2−^ and Zn^2+^ ions, stacked alternately along the c-axis (polar axis). Polar surfaces are typical of ZnO, with the basal plane being the most common. The oppositely charged ions produce positively charged Zn-(0001) and negatively charged O-(000-1) surfaces, resulting in a spontaneous polarization along the c-axis as well as a different surface energy. To maintain a stable structure, the polar surfaces generally exhibit facets or surface reconstructions, but in ZnO they are atomically flat, stable, and without reconstruction [57]. The other two most commonly observed facets for ZnO are {10–10} and {11–20}, which are non-polar surfaces and have lower energy than the {0001} facets [58]. ZnO different morphologies can be grown by tuning the growth rates along these directions. Macroscopically, a crystal has different kinetic parameters for different crystal planes, which are emphasized under controlled growth conditions. Thus, after an initial period of nucleation and incubation, a crystallite will commonly develop into a three-dimensional object with well-defined, low index crystallographic faces. It has been observed, firstly by Laudise [59], that the relationship between the velocities of ZnO crystal growth to different directions is: *v _[0001]_* > *v _[10–11]_* > *v _[10–10]_*.

**Figure 2:**
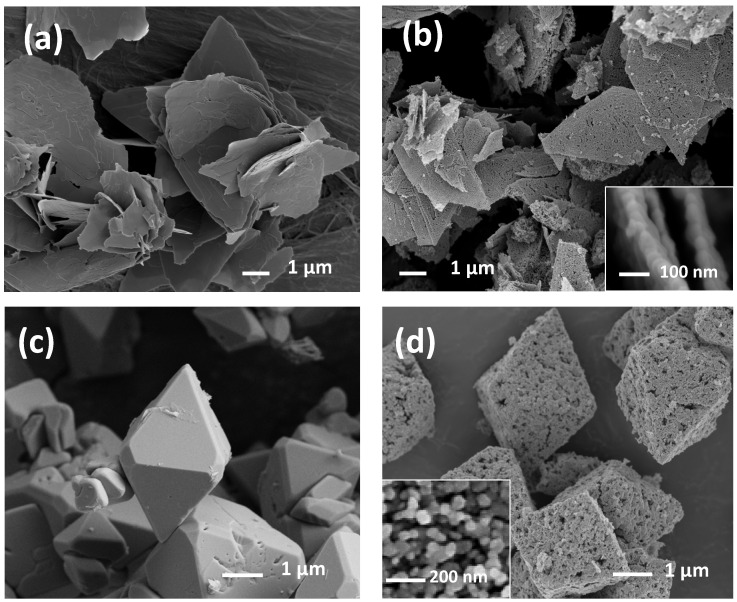
FE-SEM images of leaves precursor (**a**), leaves (**b**), bisphenoids precursor (**c**), and bisphenoids (**d**).

The Figure 2 shows the comparison between the leaves and bisphenoids precursor (samples before calcination process) and the leaves and bisphenoids after calcination. Leaves resulted from the ε-Zn(OH)_2_ direct precipitation in the synthesis solution starting from the base addition, while bisphenoids derived from the spontaneous growth of leaves kept in the synthesis solution. Leaves samples are constituted of leaves of rhombic shape, which have the longest diagonal of about 6 μm in size, while the shortest diagonal one is about 3 μm long. ZnO leaves (Figure 2b) are also characterized by a fine structure made up of nanoparticles of about 50–60 nm size. In the case of bisphenoids samples, SEM micrographs reveal an octahedral shape, which axis is about 10 µm. Again, we observe the well crystallized surfaces of bisphenoids precursor, and ZnO bisphenoids made up of nanoparticles with a size slightly smaller than that of the leaves powders. In literature, this morphology is known as octahedral [60,61], and is interpreted by us as an association of two bisphenoids ({111} and {11–1}) of original ε-Zn(OH)_2_, as described in the next section.

**Figure 3:**
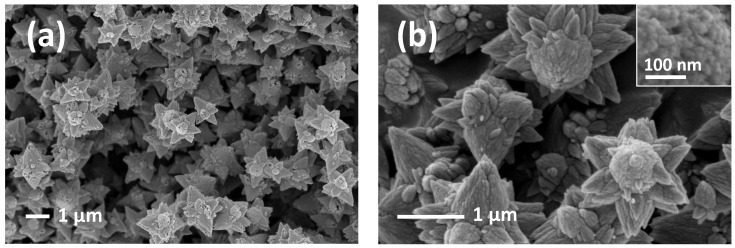
FE-SEM images of stars at different magnification (**a**,**b**).

Concerning FE-SEM analysis on the third powder (Figure 3a, b), this shows a stars-like morphology in which stars have a diameter of about 1 µm. In Figure 3b, the stars appear as if they were composed of grains about 30 nm in size. Each star is made by a central ellipsoid from which six tips radiate. This morphology reminds the 2D scheme of ZnO flowers shown in Figure 1.

**Figure 4:**
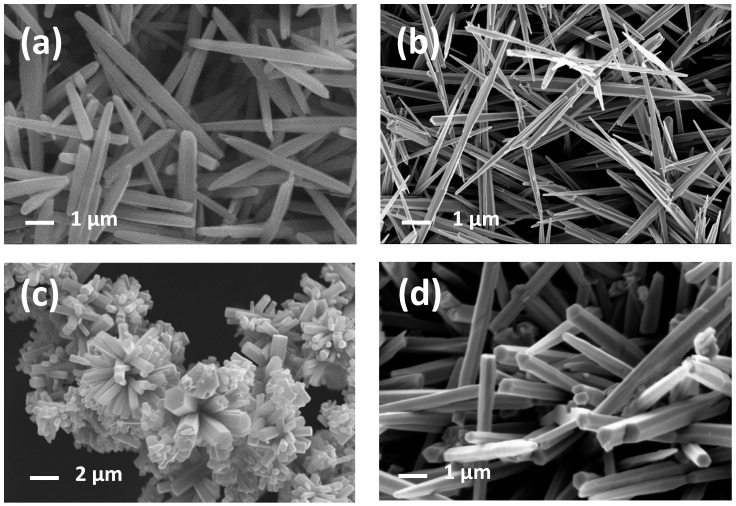
FE-SEM images of needles (**a**), long needles (**b**), flowers (**c**), and prisms (**d**).

In Figure 4, the micrographs of needles, long needles, prisms, and flowers are collected. As regards needles, they are characterized by a homogeneous distribution of elongated crystals, 200–300 nm wide and about 3–4 μm long. Long needles powders are constituted of crystals about 100 nm wide and 7–8 μm long. Hexagonal prisms about 100 nm wide represent the second last morphology. Finally, the last sample is characterized by micro flowers in which petals are hexagonal prisms, about 300–400 nm wide, and 5–6 μm long. The growth of the flower structures could be explained taking into account a possible twinning plane (80–89) that corresponds to the plane (10–11). The (80–89) twinning plane in zincite was firstly reported in Dana’s Series of Mineralogies [62]. Needles, long needles, prisms, and petals of flowers are all different crystal morphologies of ZnO. The only presence of hexagonal prisms (prisms and flowers samples) is due to the slower growth promoted by a weak base (HMTA). On the contrary, the presence of two pyramids both on the top and the bottom of the hexagonal prisms (needles and long needles samples) reveals a faster growth promoted by ammonia solution, which is a base stronger than HMTA.

Textural characterization gave specific surface areas (SSA) that can be divided into two ranges of values. Stars, needles, long needles, prisms and flowers showed a SSA under 10 m^2^/g, where the lowest is that of stars sample (5 m^2^/g). Leaves and bisphenoids exhibited a highest SSA of about 20 m^2^/g.

**Figure 5:**
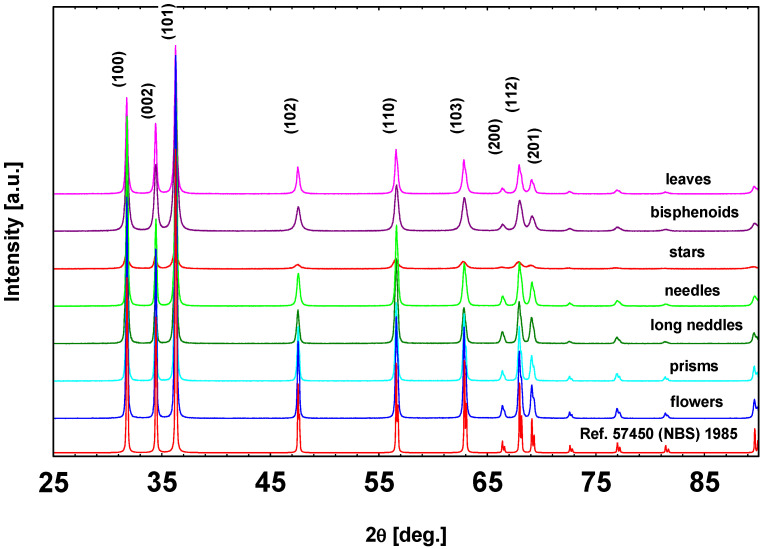
XRD patterns of the ZnO morphologies compared with that of reference.

Focusing on the structural characterization, all synthesized materials presented a hexagonal wurtzite structure (space group P6_3_mc) irrespective both on the synthesis method and the particle morphology, as shown in the XRD patterns (Figure 5). In Table 1, the unit cell parameters, the volumes, the Bragg R factors, and the crystallite sizes, evaluated by the Scherrer’s formula, are summarized. Only the crystallite sizes showed some differences. Leaves, bisphenoids and stars had the smallest crystallites with average dimension under 30 nm; long needles were characterized by an intermediate crystallite size of about 40 nm, while needles, prisms, and flowers exhibited an average crystallite dimension of about 50 nm. Considering the last four morphologies, the average crystallite sizes were apparently in disagreement with SEM images, which showed monocrystals of several hundreds of nanometers. However, the Scherrer’s formula allows to calculate the average length of the almost perfect crystals, Thereby, for needles, long needles, prisms, and flowers, the average crystallite size can be considered as an average distance between the growth defects (such as dislocations, stacking faults, etc.) due to the rapid crystallization involved in the specific synthesis methods.

### 3.2. Morphology Dependence on ZnO-Precursor

**Figure 6:**
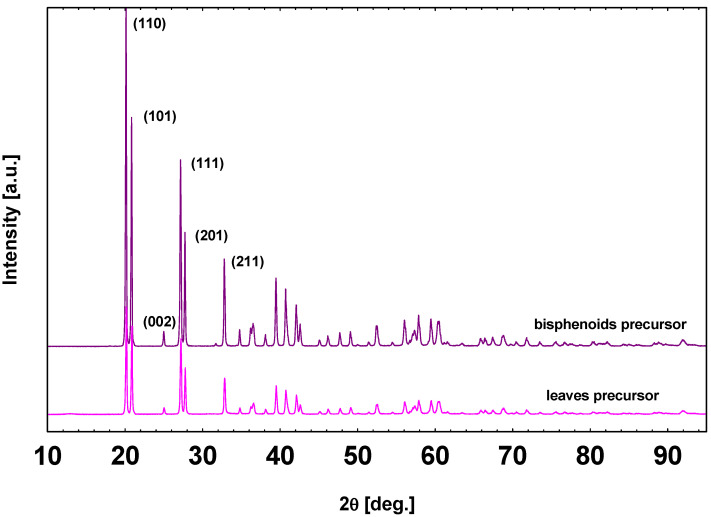
XRD patterns of the ZnO precursors (ε-Zn(OH)_2_) in form of leaves and bisphenoids.

In the case of stars, needles, long needles, prisms, and flowers, the shape is directly related to the ZnO crystalline phase through a rapid crystallization in slightly different synthesis conditions. On the contrary, as clearly observable in the Figure 2, in the cases of leaves and bisphenoids, a particularly important topic must be highlighted: they exhibit a crystalline solid precursor as an intermediate synthesis product that influences the final ZnO morphology. Leaves and bisphenoids’ solid precursor, as confirmed by SEM (Figure 2a,c) and XRD patterns (Figure 6), crystallize as orthorhombic ε-Zn(OH)_2_, space group P2_1_2_1_2_1_, parametric units cell a = 4.9050 (1) Å, b = 5.1457 (1) Å, c = 8.4780 (2) Å and volume of 214.176 (8) Å^3^ according to JCPDS Card No. 38–0385. During the calcination process, the solid precursor changes into ZnO nanoparticles through an in situ crystallization [63], releasing hydroxide ions (OH-), but maintaining the original shape and dimensions. In these cases, ZnO behaves as a pseudomorphic material: it is a crystal of a mineral that, subjected to an alteration (partial addition or partial removal of original material), changes the internal structure or chemical composition, but the external form is preserved. Such a mineral with outward crystal form of another mineral species is known as “pseudomorphism” [62]. Pseudomorphs usually are further defined according the way in which they were formed, as by substitution, encrustation, or alteration. The ZnO pseudomorphism occurs for alteration as well as for example for CaSO_4_ that becomes CaSO_4_ × 2H_2_O.

### 3.3. Morphological Analysis of the Sensing Films

**Figure 7:**
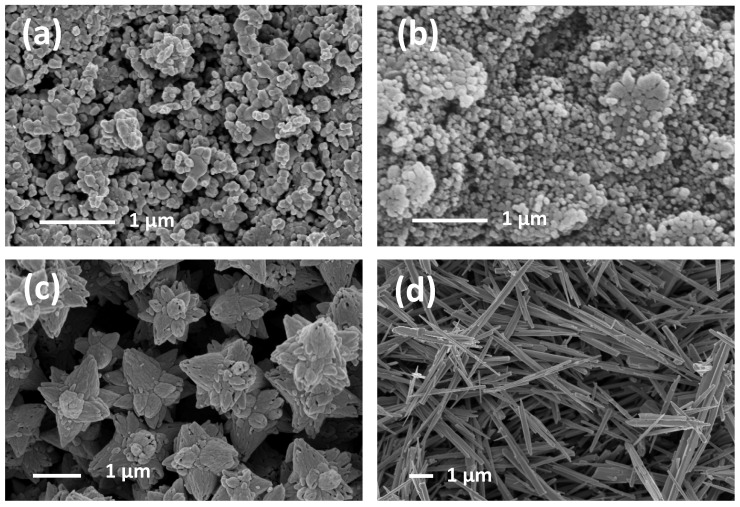
FE–SEM micrographs of the sensing films deposited starting from leaves (**a**), bisphenoids (**b**), stars (**c**), and long needles (**d**).

Morphological analysis of the sensing films after the firing process for 1 h at 650 °C, firstly highlighted two different behaviors, as shown in Figure 7: (i) leaves and bisphenoids lost their characteristic morphology and the films appeared as homogenous layers resulted from the crumbling of the nanoparticle aggregates, (ii) the other morphologies maintained their crystal shape. In the Figure 7, the examples of stars and long needles morphologies are reported. The powders constituted of stars, after calcination, looked like as grown in form of nano-particles aggregate. Surprisingly, after film firing, they revealed a different nature, i.e., a “crystallographic continuum”. In the stars structure, the crystallites were in crystallographic orientations, therefore the stars represent a first stage to become a monocrystal. However, all other powders showed a moderate grain coalescence suggesting that the firing process at 650 °C did not compromise the size of the powders obtained after calcination.

### 3.4. Electrical Characterization

In metal oxide gas sensors, the electrical properties are firstly dependent on their aptitude to modify the electrical conductivity when they come into contact with the gases of the surrounding atmosphere. On the basis of this peculiarity, a great amount of features influence the sensor characteristics. Of course, the functional material is the key constituent of the sensor, but the morphology and the size of its particles are certainly critical factors to determine its detection properties [64]. In particular, morphology and size influence the specific surface area and consequently the surface charge density, which is a function closely connected with the reception property of the sensor itself [65]. Besides that, many other factors are crucial for gas sensor performance, being the first one, the fabrication procedure (thin or thick film) and correspondently porosity, particle agglomeration, sintering control, etc. All these features together with the functional material affect the transduction function, which in turn determines the ability of the sensor to modify its conductivity in presence of the test gases.

**Figure 8:**
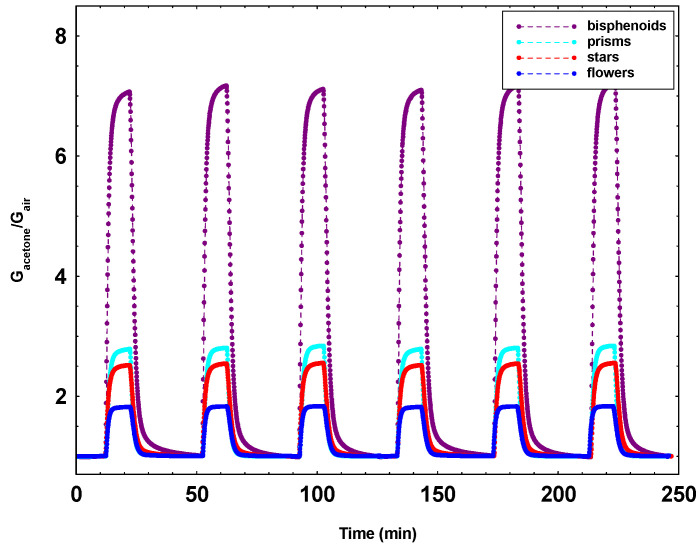
Dynamical responses to 0 and 1 ppm of acetone alternately for a bisphenoid’s, a star’s, a prism’s, and a flower’s sensors (operation temperature of 450 °C).

Apart from the aptitude of the specific morphologies to detect the various gases, it is however of the utmost importance to verify for all samples the electrical stability and repeatability. Indeed, they are essential characteristics in order to obtain efficient and reliable sensors able to operate continuously for long time. In Figure 8, an experiment on a bisphenoid’s, a star’s, a prism’s, and a flower’s sensors alternately subjected to 0 and 1 ppm of acetone is reported. This test highlights the good repeatability of the sensors, confirming that the screen-printing technology together with a proper powder preparation permit to fabricate thick film gas sensors with optimal electrical properties [53].

Other features of screen-printing technology, such as the high firing temperature or the chance to fabricate in one batch up to 500 sensors, are conditions that contribute to assess screen-printed MOX gas sensors highly reliable among the various kinds of chemical gas sensors.

**Figure 9:**
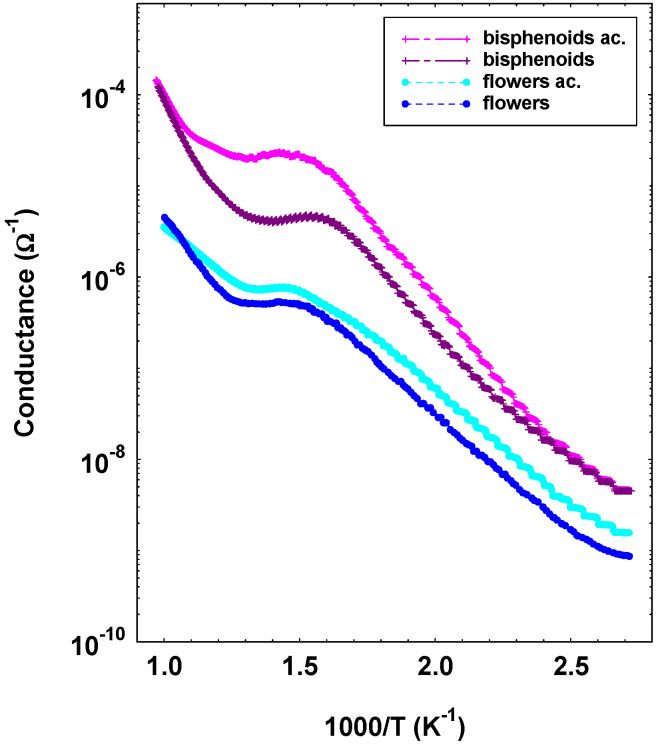
Conductance as a function of temperature in dry air and in a mixture of dry air and acetone (10 ppm) for bisphenoids and flowers sensors.

Considering the specific electrical characterizations, an Arrhenius plot, which consists of the measurement of the conductance as a function of temperature in different atmospheres, was carried out. In this work, dry air and a mixture of dry air and acetone (10 ppm) were chosen, for comparison. Such measurement highlighted for all samples a n-type semiconductor behavior, the conductivity exhibiting a trend with temperature divided in more ranges, behavior ascribable to different chemical adsorption phenomena. Such particular trend is typical of MOX gas sensors and justified by the formation of inter-grain Schottky barriers that modulate the film conductivity [24]. Moreover, such measurement gives information on the magnitude of conductivity and a preliminary study of the response toward the tested gas. For the sake of clarity, in Figure 9, it has been chosen to report the measurements relating to the sensors showing the behavior with the greatest difference of all the samples. Specifically, the conductance as a function of the inverse of temperature of a bisphenoids and a flowers sample are reported, the other samples positioning in between. Two main observations can be made: (i) concerning the conductivity, it resulted higher in the thick films composed of aggregates of nanoparticles with respect to the ones in form of crystals, a phenomenon probably due to the different inter-particles percolation paths; (ii) with respect to the difference between the measurement in air or in 10 ppm of acetone in air, it resulted greater for the bisphenoids sample than for the flower sample. Since acetone is a reducing gas, the result is a clear indication of the greater response capacity toward acetone of the bisphenoids sample than for the flowers one.

**Figure 10:**
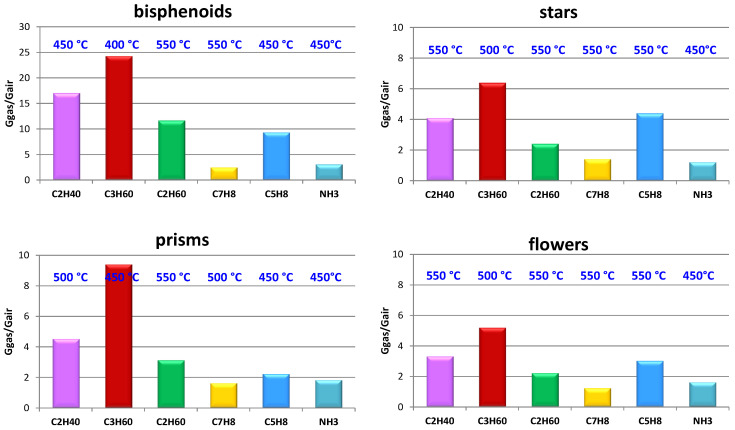
Responses toward 10 ppm of acetaldehyde, acetone, ethanol, toluene, isoprene, and ammonia in dry air for the sensors based on bisphenoids, stars, prisms, and flowers.

Passing to dynamical measurements of conductance in presence of the tested gases (acetaldehyde C_2_H_4_O, acetone C_3_H_6_O, ethanol C_2_H_6_O, toluene C_7_H_8_, isoprene C_5_H_8_, and ammonia NH_3_), the responses toward each tested gas at the concentration of 10 ppm in dry air, for the sensors based on bisphenoids, stars, prisms, and flowers are reported in Figure 10. Such response values are calculated as mean values of couples of nominally identical sensors, and they are moreover measured at the operating temperature for which the response resulted maximized, as shown in the figure for each sensor. The results of the Figure 10 are relative to the sensors that showed the best performance (bisphenoids), the worst (flowers), while the based-prisms sensors are shown as example of crystals with peculiar electronic characteristics, as reported in a previous work of some of the authors [54]. Finally, the stars-based sensors have been chosen as a case of only apparent nano-particle aggregate. The results of Figure 10 are in good agreement with the morphological, textural, and structural characterizations. In this context, particular attention shall be paid to the evolution of crystallites as grown until their transformation throughout all the cycle of preparation of thick films. Indeed, in the case of leaves and bisphenoids, the crystallites agglomerate to form nanometric roundish grains proportionally sized to that of crystallites, the grains moderately coalescing under firing. As described above, differently from nano-particle aggregates, needles, long needles, prisms, and flowers resulted monocrystals in which the crystallites must be considered only as the regions of almost perfect crystal periodicity, while in stars morphology, the crystallites constitute a “crystallographic continuum” i.e., the first stage of a monocrystal. Due to such characteristics, the crystallite dimensions of the crystalline samples cannot be directly connected with the sensors performances. For this purpose, it resulted instead as more appropriate to compare the crystalline morphologies as offered by SEM observations.

Going back to the electrical measurements, it can be observed in the Figure 10 that all the sensors showed the best responses toward few gases: acetone, acetaldehyde, and isoprene, the morphology not giving particular aptitude for a specific gas.

The responses at 10 ppm of acetone, which is certainly the gas offering the largest responses, are summarized in the Table 2 for all the tested temperatures. It resulted that the sensors prepared with powders constituted of nanoparticle aggregates in form of bisphenoids and leaves showed the highest responses. In particular, bisphenoids exhibited better performance and also smaller grains with respect to leaves, the latter offering larger grains and correspondently worse performance, according to [32]. On the other hand, all the sensors prepared with nanocrystals resulted less able to detect the reducing tested gases. With respect to the test in acetone, the maximum response values resulted in decreasing order for long needles, needles and flowers, respectively. Even in the case of crystal shaped samples, the value of the gas response is inversely correlated to the size of the crystal, in agreement with the SEM micrographs carried out on the thick films (see Figure 7). Such difference of performance between sensors based on nanoparticle aggregates and monocrystals was already described and interpreted for few types of crystals [34,54]. The same result has been now demonstrated valid for a large number of crystalline morphologies, including the particular case of stars, which was shown to be an apparent case of nano-crystal aggregate.

**Figure 11:**
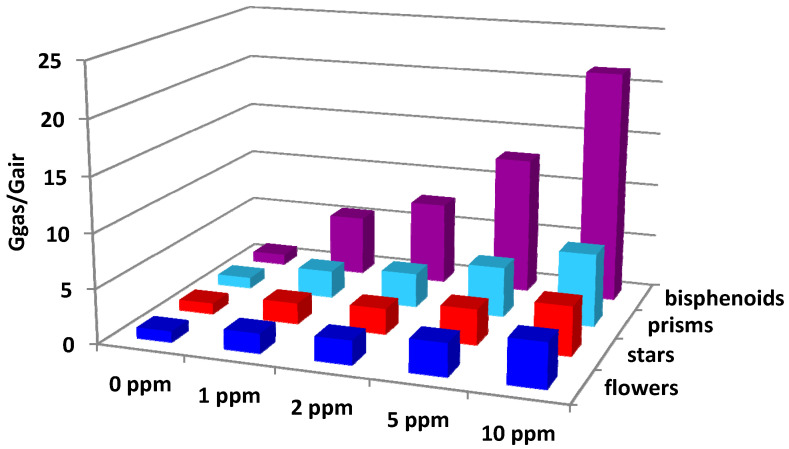
Responses to 10, 5, 2, and 1 ppm of formaldehyde at the operating temperature of 500 °C for flowers, stars, prisms and bisphenoids sensors.

Moreover, in addition to the other tested gases, ZnO sensors underwent to formaldehyde, a particularly dangerous compound for human health, to verify if they were suitable to detect it at low concentrations. In the Figure 11, the responses at 10, 5, 2, and 1 ppm of formaldehyde at the operating temperature of 500 °C for the selected ZnO sensors are reported. The result was very attractive, so appearing as possible the formaldehyde sub-ppm detection.

In addition to the magnitude of the gas response, in gas sensor field, response and recovery times are also important parameters. With reference to the Figure 8, defining the response time as the time necessary to reach 90% of the response value and similarly for the recovery time, the values calculated for bisphenoids sensors resulted 2.3 min for the response time and 4.4 for the recovery one. In the case of prisms, the times resulted as lower: 1 min for the response time and 2.2 for the recovery time. This is reasonable due to the lower number of contacts between the particles, and further, in the case of prisms, constituted by well-defined and smoothed surfaces.

However, differently from what usually believed, the sensor time parameters are mainly driven by the geometry of the test chamber, in particular by the volume, rather than by the physical-chemical characteristics of the sensor itself. Indeed, the smaller the test chamber volume, the shorter the response and recovery times [66].

## 4. Conclusions

An extensive study on the synthesis methods, the growth mechanisms, and the sensing properties of seven ZnO powders with different morphologies was carried out, obtaining the following main results.

The ZnO powders were successfully obtained with optimized routes, starting from the same reagents in water solution by changing the synthesis conditions. Two classes of morphologies were obtained: the nanoparticles’ aggregates (leaves and bisphenoids) and the monocrystals based ones (stars, needles, long needles, prisms, and flowers). Their growing mechanisms were systematically analyzed highlighting two main ways: (i) the formation of a solid precursor (ε-Zn(OH)_2_) that changes into ZnO through an in situ crystallization obtaining aggregates of nanoparticles; (ii) a rapid crystallization in growth solution for monocrystal based morphologies. The corresponding sensing layers were studied with particular interest to their gas sensing properties toward a wide number of reducing gases in relation to the functional material morphology and the respective growing mechanism.

It turned out, similarly to powders, there were two different behaviors. The samples constituted by nanoparticle aggregates exhibited the best performances toward all tested gases with respect to monocrystals based sensors, that, albeit with some difference, all showed worsened performances. Due to the large number of investigated forms, this result confirms that, almost for reducing gases, the three-dimensional shape, like a spherical particle, is the most suitable form in the MOX chemiresistive sensing process.

The present work permitted to select sensors with optimal sensing properties (sub-ppm acetone and formaldehyde detection), exploiting ZnO aptitude to grow in different forms. At the same time, the synthesis involved is easy to do, not expensive, and further chemical processes are not necessary. Moreover, this strategy opens the way for future applications in other functional material preparation.

## Figures and Tables

**Table 1:** Crystalline phase, the unit cell parameters values: a, c, and volume, Bragg R factor, and crystallite size for all ZnO morphologies. In parentheses, the estimated standard deviations are referred to the last digit.

Sample	Structure	a (Å)	c (Å)	Vol. (Å^3^)	R_B_	Crystallite Size
Leaves 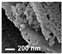	Wurtzite (P6_3_mc)	3.24899 (3)	5.20664 (8)	47.598 (1)	2.65	26
Bisphenoids 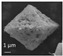	Wurtzite (P6_3_mc)	3.25018 (3)	5.20819 (8)	47.647 (1)	1.76	24
Stars 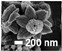	Wurtzite (P6_3_mc)	3.25262 (5)	5.21199 (13)	47.753 (1)	2.41	29
Needles 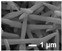	Wurtzite (P6_3_mc)	3.25012 (2)	5.20789 (7)	47.642 (1)	2.18	48
Long needles 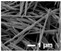	Wurtzite (P6_3_mc)	3.25081 (2)	5.20918 (7)	47.674 (1)	2.63	39
Flowers 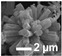	Wurtzite (P6_3_mc)	3.24988 (2)	5.20624 (4)	47.620 (1)	2.70	49
Prisms 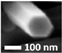	Wurtzite (P6_3_mc)	3.25064 (3)	5.20776 (7)	47.656 (1)	3.39	49

**Table 2:** Values of sensors responses toward acetone (10 ppm, dry air) as a function of the sensors operating temperature.

Sensors Responses G_acetone_/G_air_		350 °C	400 °C	450 °C	500 °C	550 °C
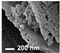	Leaves	8.6	13.4	21.4	19.6	7.2
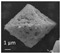	Bisphenoids	17.0	26.4	23.9	19.9	5.5
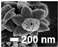	Stars	3.5	4.9	6.4	7.7	10.5
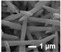	Needles	4.8	5.3	6.4	10.6	5.1
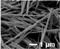	Long needles	5.0	5.4	6.5	11.0	5.5
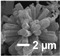	Flowers	4.3	4.8	5.6	9.4	7.3
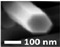	Prisms	6.3	8.5	11.9	13.0	11.5

## Data Availability

All data are included in the paper.
